# Transesophageal Echocardiography Guided Partial Right Atrial Inflow Occlusion - A Case Study

**DOI:** 10.24908/pocus.v7i2.15663

**Published:** 2022-11-21

**Authors:** Lucas Barboza, Rob Tanzola

**Affiliations:** 1 Queen's School of Medicine, Queen's University Kingston, Ontario Canada; 2 Department of Anesthesiology and Perioperative Medicine, Queen's University Kingston, Ontario Canada

**Keywords:** Right atrial inflow occlusion, Transesophageal echocardiography, transient hypotension, thoracic endovascular aneurysm repair, balloon occlusion

## Abstract

Deployment of stent-grafts and other endovascular devices is a common technique for various vascular repair procedures. Induced, transient, periods of hypotension are essential to the precise deployment of a device as this minimizes displacement that can result from high pressure aortic flow. Partial inflow occlusion of the right atrium is a reliable, precise, and safe method of achieving this. We present a case where intraoperative transesophageal echocardiography (TEE) was used to guide and confirm balloon placement for right atrium inflow occlusion during a thoracic endovascular aneurysm repair (TEVAR) procedure for repair of an aortic dissection in a 67 year old male. This highlights a novel use of TEE in the context of endovascular surgery, and showcases an alternative method of reliably achieving transient hypotension.

## Case Description

A 67 year-old male presented to the emergency department with sudden onset tearing central chest pain, radiating along his left sternal edge. The patient was alert and oriented and not in acute distress, with triage vitals of BP 174/107, HR 76, RR16, SpO_2_ 99% on room air, T36.7ºC and pain that had subsided to 2/10 on the Numeric Rating Scale (NRS) from an initial 6/10 at onset. He had a medical history notable only for hypertension, controlled with Ramipril. He was assessed in the Emergency Department where a chest X-ray, CBC, troponin, D-Dimer, 12 lead ECG and CT scan of the chest were performed. CT scan of the chest identified a small dissection of the distal aortic arch and proximal descending aorta distal to the origin of the left subclavian artery, measuring 4.2 cm in maximal diameter (Debakey Type IIIa, Stanford Type B). All other investigations were unremarkable. A repeat scan 1 month later revealed expansion of the dissection, indicative of a slow-growing focal intimal tear or pseudoaneurysm, prompting an urgent vascular surgery consult. The patient had been asymptomatic since the initial episode. The patient was determined to be a candidate for a left carotid subclavian bypass and thoracic endovascular aneurysm repair (TEVAR). Left carotid to subclavian bypass is commonly performed with TEVAR to ensure adequate blood flow to the left subclavian which can be compromised by the proximal landing zone of the stent graft.

**Figure 1:**
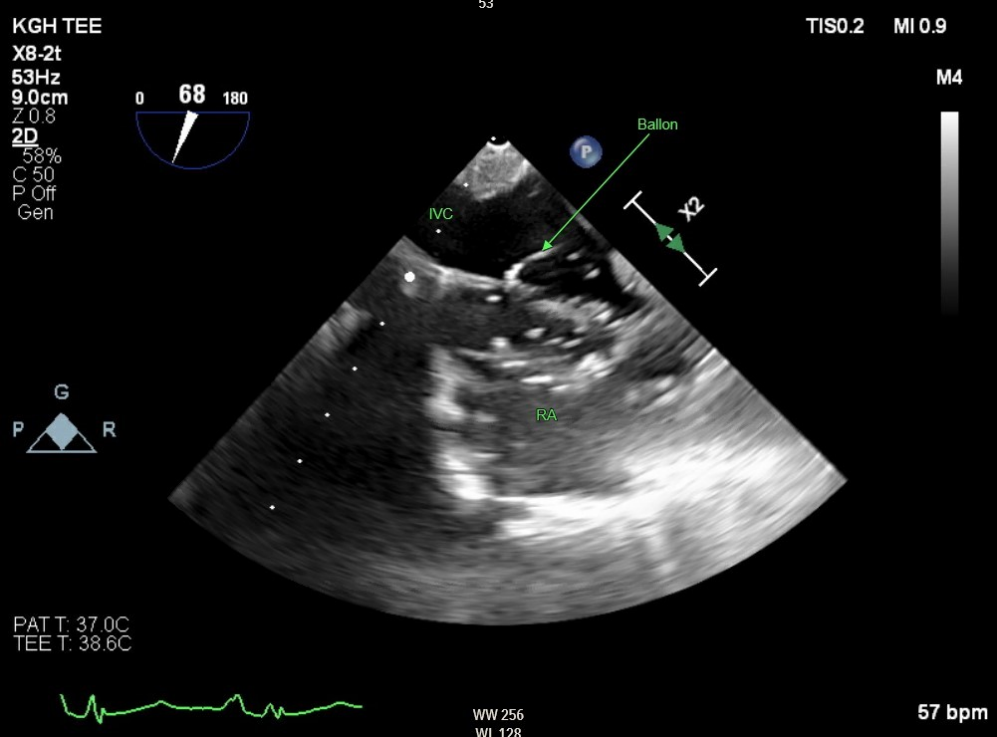
A transesophageal modifiedbicaval view demonstrating the inflow occlusion balloon inflated and sitting in the mid right atrium. IVC = inferior vena cava, RA = right atrium.

**Figure 2:**
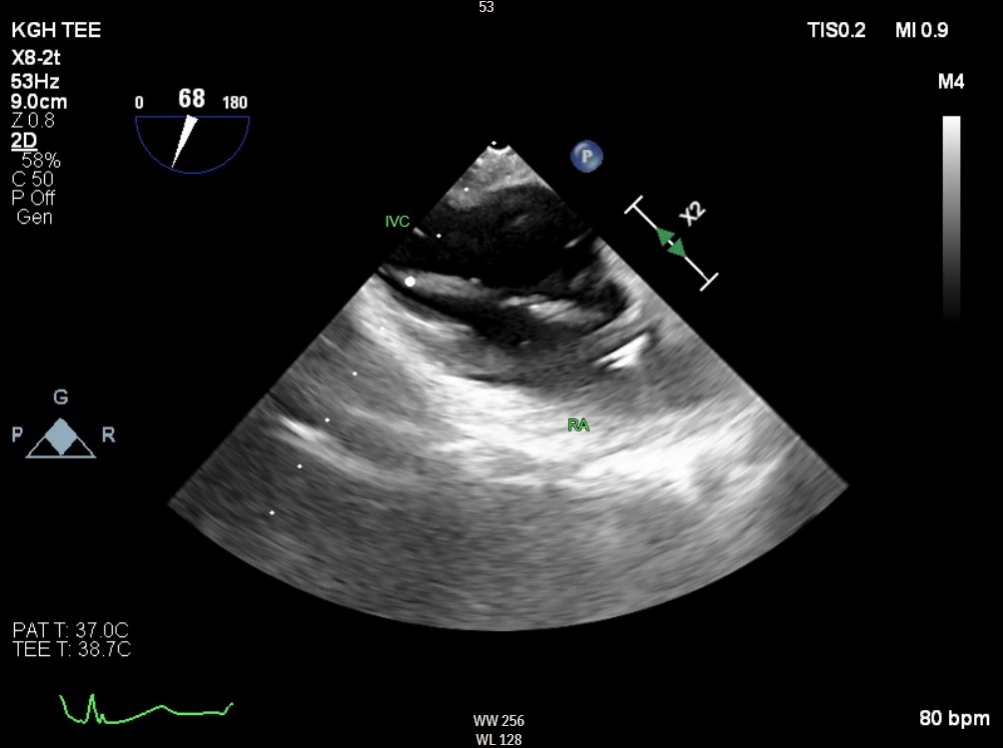
A transesophageal modified bicaval view demonstrating traction on the balloon catheter causing occlusion of the right inferior vena cava orifice.

The patient was brought to the operating room where general anesthesia was induced and endotracheal intubation was performed. An arterial line was placed in the radial artery and a TEE probe was inserted. The procedure started with a successful carotid subclavian bypass using a 6 mm Dacron graft. Next the patient was positioned for the TEVAR procedure and access was obtained through bilateral femoral arteries. During the TEVAR procedure, the technique of partial inflow occlusion using a 32 mm long Coda balloon catheter (Cook Incorporated, Bloomington, IN) was performed as a means of achieving transient hypotension for graft deployment. This less commonly used method of inducing hypotension offered a reliable alternative to transvenous pacing and pharmacological methods, with the unique utilization of TEE to confirm balloon placement. The midesophageal bicaval TEE view offered a clear visualization of accurate balloon placement in the right atrium (Figure 1). The balloon was then inflated to 32 mm as a test run. Once traction was applied to the balloon by the operator, confirmation of right atrial inflow occlusion was also well described with TEE imaging (Figure 2, online Video S1). Systolic blood pressure rapidly dropped to approximately 60 mmHg and a single 32 x 200 mm graft was deployed successfully during this period of occlusion. The balloon was then promptly deflated and removed without complication. Finally, an Amplatz plug was deployed for embolization of the proximal subclavian artery. There were no perioperative complications, and the patient was discharged on post-operative day 3 with follow up CT scans scheduled. The patient did make note of some hoarseness postoperatively but was generally satisfied with the treatment provided.

 

## Discussion

The ability to induce a rapid, reversible period of systemic hypotension has become an important component of many endovascular repair procedures. Precise positioning when deploying stent-grafts or other endovascular devices may be impaired when exposed to high pressure, pulsatile blood flow from the aorta as the cardiac output can move the device. Temporarily reducing cardiac output and systolic blood pressure helps to minimize these disruptions and ensure accurate placement of the device. Some common procedures that benefit from this technique of induced transient hypotension include thoracic TEVAR, transcatheter aortic valve implantation (TAVI), and cerebral aneurysm clipping.

There are a variety of techniques that may be used to achieve this temporary hypotension, each having advantages and disadvantages. Pharmacological methods are the most common modality used to either induce temporary asystole or systemic hypotension in a non-invasive manner. Most commonly adenosine is used. However, doses must be individualized to the patient and can often be overshot or responses may be unpredictable in both hypotensive effect and duration. This can lead to a sudden, unexpected reversal of hypotension before the procedure is complete, and less flexibility to prolong the procedure should more time be needed 1. Restoration of normotension is slower when using pharmacological means compared to other techniques, increasing risk of complications. There also remain relative contraindications to adenosine use, including asthmatic patients (due to bronchoconstrictive effects), patients with advanced coronary artery disease, and patients with 2^nd^ or 3^rd^ AV block.

Rapid ventricular pacing via a temporary transvenous pacing wire is a technique used to disrupt atrioventricular synchrony, ventricular filling, and cardiac output. This allows a more precise induction and reversal of hypotension and can be adjusted to the exact desired length of the procedure. However, there remains a risk of inducing sustained arrhythmias and increased myocardial oxygen demand due to the tachycardia, as well as the risks of placing the central line and wire 2. This technique would also be limited to those with experience placing a temporary pacer wire.

Partial inflow occlusion of the inferior vena cava (IVC) using a balloon was used in this case as pharmacologic means were deemed to be too unpredictable and the expertise to provide transvenous pacing were not reliably available. Partial inflow occlusion of the IVC offers reversible, rapid, and highly adjustable periods of hypotension. The technique uses a balloon catheter inserted in the common femoral vein, and advanced into the right atrium under fluoroscopic or transesophageal echocardiogram (TEE) guidance. There it is inflated with a contrast-saline mixture until the desired balloon diameter is achieved. The balloon catheter is then positioned under traction to occlude inflow of the IVC until the desired hypotensive effect is achieved. A target mean systolic pressure of 50 mmHg can be reliably achieved within approximately 45 seconds, and once the procedure is complete the balloon is simply deflated and removed, with return to normotension within 1-2 minutes 3.

Similar to pacing, this technique avoids the unpredictable duration and effect that comes with using pharmacological methods. However, an additional advantage is that balloon occlusion does not increase cardiac oxygen demand as in rapid pacing, allowing for optimal use in patients with ischemic heart disease 3. Occlusion can be maintained for just as long as required by the surgeon, should any delay in device deployment arise. It is notable that certain pathologies which interfere with balloon placement may exclude partial inflow occlusion as a viable technique. For example, a large, remnant Eustachian valve or a Chiari network can cause a physical barrier to endovascular placement of the balloon or a pacer wire in the right atrium 4, 5. Assessment of the right atrium in both the midesophageal 4-chamber and the bicaval views can adequately reveal these anatomic variants. Visualization of the IVC-right atrial junction will demonstrate the size of orifice that need to be occluded and will confirm the appropriate placement of the balloon. As the size of the IVC is generally less than 25 mm, the 32 mm Coda balloon is likely adequate in most patients. A 40 mm balloon is also available. TEE also provided post-procedure assessment for pericardial effusion which is prudent whenever intracardiac catheters and devices are used. 

Our case study highlights the utility of using TEE for accurate and reliable visualization of balloon placement during the technique of right atrial inflow occlusion. TEE guidance of right atrial inflow occlusion would be advantageous in cases where fluoroscopy is not available or where sparing additional contrast and radiation exposure is desired. Additionally, it may serve as a confirmatory modality if there is a question about the appropriate position of the occlusion balloon. The point of care use of TEE in this case report showcases the utility of echocardiography in the perioperative setting.

## Patient Consent and Ethics Approval

The authors gained consent from the patient to publish, as well as ethics approval. 

## Disclosures

None.

## Supplementary Material

 Video S1 A transesophageal modified bicaval view demonstration localization balloon in the right atrium (RA). It is subsequently inflated and with traction it is pulled distally causing occlusion of inferior vena cava (IVC) inflow at the level of the RA-IVC junction.
